# Ir- and Pt-Doped InTe Monolayers as Potential Sensors for SF_6_ Decomposition Products: A DFT Investigation

**DOI:** 10.3390/ma18174022

**Published:** 2025-08-28

**Authors:** Juanjuan Tan, Shuying Huang, Jianhong Dong, Jiaming Fan, Dejian Hou, Shaomin Lin

**Affiliations:** 1 School of Chemical and Environmental Engineering, Hanshan Normal University, Chaozhou 521041, China; 20190029@hstc.edu.cn (J.T.); 202301017232@stu.hstc.edu.cn (S.H.); 202201017205@stu.hstc.edu.cn (J.F.);; 2 School of Materials Science and Engineering, Hanshan Normal University, Chaozhou 521041, China

**Keywords:** InTe monolayer, Ir and Pt doping, SF_6_ decomposition products, gas sensing, adsorption mechanism

## Abstract

The burgeoning demand for reliable fault detection in high-voltage power equipment necessitates advanced sensing materials capable of identifying trace sulfur hexafluoride SF_6_ decomposition products (SDPs). In this work, the first-principles calculations were employed to comprehensively evaluate the potential of Ir- and Pt-doped InTe (Ir-InTe and Pt-InTe) monolayers as high-performance gas sensors for the four specific SDPs (H_2_S, SO_2_, SOF_2_, SO_2_F_2_). The results reveal that Ir and Pt atoms are stably incorporated into the hollow sites of the InTe monolayer, significantly reducing the intrinsic bandgap from 1.536 eV to 0.278 eV (Ir-InTe) and 0.593 eV (Pt-InTe), thereby enhancing the material’s conductivity. Furthermore, Ir-InTe exhibits selective chemisorption for H_2_S, SO_2_, and SOF_2_, with adsorption energies exceeding −1.35 eV, while Pt-InTe shows chemisorption capability for all four SDPs. These interactions are further supported by significant charge transfer and orbital hybridization. Crucially, these interactions induce notable bandgap changes, with Ir-InTe showing up to a 65.5% increase (for SOF_2_) and Pt-InTe showing an exceptional 105.2% increase (for SO_2_F_2_), alongside notable work function variations. Furthermore, recovery time analysis indicates that Ir-InTe is suitable for reusable H_2_S sensing at 598 K (0.24 s), whereas Pt-InTe offers recyclable detection of SO_2_ (5.27 s) and SOF_2_ (0.16 s) at the same temperature. This work provides theoretical guidance for the development of next-generation InTe-based gas sensors for the fault diagnosis in high-voltage power equipment.

## 1. Introduction

Sulfur hexafluoride (SF_6_), as an excellent gas insulation medium owing to its superior dielectric strength and arc quenching capabilities, is extensively used in high-voltage power equipment (HVPE), including circuit breakers, switchgear, and gas-insulated substations [1,2]. However, under some extreme conditions such as high electric fields and partial discharges, SF_6_ inevitably decomposes into a series of toxic byproducts (H_2_S, SO_2_, SOF_2_, and SO_2_F_2_) [3,4]. These SF_6_ decomposition products (SDPs) can accelerate the aging and degradation of insulating materials, potentially leading to a decline in the insulation performance of high-voltage electrical equipment and ultimately causing failure or catastrophic breakdown [5,6]. Fortunately, these SDPs can serve as early indicators of incipient faults within the equipment [7]. To enable early warning and online monitoring of SDPs, researchers have actively pursued the development of high-performance gas sensing materials. However, despite the application of online monitoring technologies such as semiconductor metal oxide sensors [8], they face significant challenges, including insufficient sensitivity, poor selectivity, and relatively high operating temperatures [9]. Particularly in complex field environments, achieving highly selective detection of trace SDPs remains a significant and unresolved challenge.

In recent years, two-dimensional (2D) materials have demonstrated unparalleled advantages in gas sensing due to their enormous specific surface area, abundant surface active sites, and tunable electronic structures [10]. Various types of 2D materials have been extensively investigated for detecting SDPs [11,12]. For instance, graphene and its functionalized derivatives exhibit excellent adsorption and response capabilities towards gases such as SO_2_ and H_2_S, attributed to their high conductivity and introduced surface functional groups [13,14]. Transition metal dichalcogenides (TMDs), including MoSe_2_ and SnS_2_, have proven effective in adsorbing and detecting specific SDPs through modulation of their electronic structures and defect states [15]. Emerging 2D materials, such as MXenes [16,17], 2D oxides [18], nitrides [12,19], and GeSe monolayer [20], have also demonstrated sensitivity to particular SDPs. Recent theoretical works continue to validate the DFT approach by screening novel materials, such as Ag-doped graphdiyne, for their potential in SDP sensing [21]. Currently, indium ditelluride (InTe), a novel 2D semiconductor with an appropriate bandgap and distinctive structural characteristics, offers a promising platform for constructing next-generation sensors. However, pristine InTe monolayers predominantly exhibit weak physical adsorption towards SDPs [22]. Such feeble interactions typically result in low adsorption energies and minimal charge transfer [23], which fail to induce significant changes in the electrical signals of the substrate material. Consequently, these limitations restrict the sensitivity and recovery speed of InTe-based gas sensors, hindering their ability to meet practical requirements for rapid and precise detection of trace SDPs.

To address the inherent limitations of pristine InTe monolayers, surface modification, particularly the introduction of metal atom doping, offers an effective strategy to alter the material’s local electronic structure and surface activity. Extensive research has confirmed that single-metal atom doping can significantly enhance the adsorption capacity and sensing response of various two-dimensional materials, such as graphene [13], MoS_2_ [6], BN [24], and InN [25], towards SDPs. For example, doping with noble metals like Pt, Pd, and Au can leverage their unique electronic structures and catalytic activities to significantly enhance the gas sensitivity of 2D materials towards target gases [2,3,26,27]. Similarly, doping with transition metals such as Fe, Co, and Ni may facilitate stronger coordination or chemical bonding through interactions between their d-orbitals and the p-orbitals or π bonds of gas molecules [4], consequently increasing adsorption energy and charge transfer efficiency [28]. Considering Ir and Pt as representative transition and noble metals, respectively, they possess unique d-orbital electron configurations and catalytic activities. These characteristics are anticipated to significantly enhance the adsorption capacity of the InTe monolayer towards SDPs by modifying its local electronic structure.

Here, first-principles calculations are employed to systematically investigate the adsorption behaviors and sensing properties of Ir- and Pt-modified InTe (denoted as Ir-InTe and Pt-InTe) monolayers towards four typical SDPs (H_2_S, SO_2_, SOF_2_, SO_2_F_2_). The stability of the doped InTe monolayers are first assessed by evaluating their binding energies and electronic structures. Subsequently, the optimal adsorption configurations are identified by comparing the adsorption energies of the SDPs on both Ir-InTe and Pt-InTe surfaces. The microscopic interaction mechanisms between the target gases and the substrates are elucidated through analyses of the electronic properties, including projected density of states, charge density distribution, and differential charge density. Furthermore, the bandgap, work function, and recovery time are calculated for each adsorption system to theoretically assess the feasibility of Ir-InTe and Pt-InTe monolayers as SDP sensors and to explore their potential sensing mechanisms. This work provides important theoretical guidance for the design and development of novel high-performance InTe-based gas sensors, and the proposed sensors may reduce emissions of SF_6_, a potent greenhouse gas, by reducing equipment failures. This paper is organized as follows: Section 2 details the computational methods employed in this study. Section 3 presents and discusses the results regarding the material properties and gas sensing performance. Finally, Section 4 summarizes the key findings and conclusions of our investigation.

## 2. Computational Details

All theoretical calculations were performed using the DMol^3^ module of the Materials Studio 2020 software package (Dassault Systèmes BIOVIA, San Diego, CA, USA) [29]. The exchange-correlation functional was described by the Peered–Burke–Ernzerhof (PBE) functional within the generalized gradient approximation (GGA) [30,31], and van der Waals interactions were included using the Tkatchenko–Scheffler (TS) method [32]. Electron–ion interactions were modeled using DFT semi-core pseudopotentials (DSPP), which explicitly treated the valence and semi-core electrons [33]. Valence electron wave functions were expanded using the high-precision double numerical plus polarization (DNP) basis set [34], with a cutoff radius of 5.0 Å. A 4 × 4 × 1 supercell model of single-layer InTe was constructed, incorporating a 20 Å vacuum layer perpendicular to the monolayer to eliminate spurious interactions between periodic images. Brillouin zone integration was performed using the Monkhorst–Pack method [35]. A 5 × 5 × 1 K-point mesh was employed for geometric optimization, while a denser 10 × 10 × 1 mesh was used for calculating electronic properties. Convergence criteria for geometry optimizations were set as follows: total energy change < 1.0 × 10^−5^ Ha, maximum force on each atom < 0.002 Ha/Å, and maximum atomic displacement < 0.005 Å. Self-consistent field (SCF) calculations were considered converged when the total energy change was less than 1.0 × 10^−6^ Ha.

The binding energy (*E*_bin_) was computed in order to assess the post-modification structural stability of Ir-InTe and Pt-InTe monolayers [36]:

(1) $$E_{\mathrm{bin}\;}=\;E_{\mathrm{TM}-\mathrm{InTe}}-E_{\mathrm{InTe}}-E_{\mathrm{TM}}$$

Transition metal (TM)-modified InTe, pristine InTe, and an isolated TM atom are denoted by the symbols $E_{\mathrm{TM}-\mathrm{InTe}}$, $E_{\mathrm{InTe}}$, and $E_{\mathrm{TM}}$ respectively, along with their corresponding total energies. According to recognized DFT benchmarks, *E*_bin_ values larger than −3.0 eV indicate thermodynamically stable doping. Additionally, the adsorption affinity of TM-InTe monolayers for gas molecules was assessed using the adsorption energy (*E*_ads_), which is defined as [37]:

(2) $$E_{\mathrm{ads}}\;={\;E}_{\mathrm{gas}\text{@}\mathrm{TM}-\mathrm{InTe}}-E_{\mathrm{TM}-\mathrm{InTe}}-E_{\mathrm{gas}}$$

where $E_{\mathrm{gas}\text{@}\mathrm{TM}-\mathrm{InTe}}$ and $E_{\mathrm{gas}}$ represent the energies of the TM-InTe with an adsorbed gas and a free gas molecule, respectively. A more negative *E*_ads_ indicates greater stability of the adsorption process. Moreover, the adsorption stability can also be qualitatively predicted via charge transfer (*Q*_T_) between the gas and adsorbent, expressed as [38,39] *Q*_T_ = *Q*_i_ − *Q*_j_, where *Q*_i_ and *Q*_j_ are the charges of the adsorbed and isolated gas molecules, respectively. The negative *Q*_T_ indicates that the adsorbed gas molecule acquires electrons from the substrate.

## 3. Results and Discussion

### 3.1. Properties of Ir-InTe and Pt-InTe Monolayers

**Figure 1:**
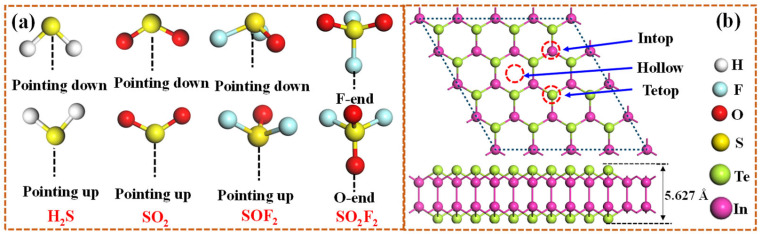
Atomic configurations of (**a**) four SDPs molecules and (**b**) pristine InTe monolayer after geometry optimization; the three possible doped sites are also given in (**b**).

**Figure 2:**
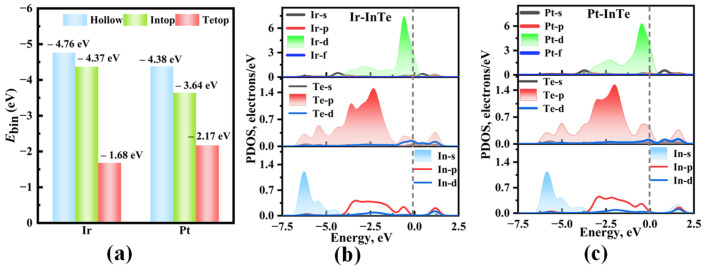
(**a**) Binding energies of Ir and Pt atoms at various doping sites in InTe monolayer, along with the projected density of states (PDOSs) of the most stable (**b**) Ir-InTe and (**c**) Pt-InTe monolayers.

As shown in Figure 1, the hexagonal InTe monolayer is typically characterized by a Te-In-In-Te stacking sequence, where two inner hexagonal planes of In atoms are covalently bonded to each other and are further sandwiched between two outer hexagonal planes of Te atoms. Electronically, the single-layer InTe is predicted to be an indirect semiconductor with the calculated bandgap and lattice parameter of 1.536 eV and 4.388 Å, which matches well with the previous result (1.44 eV) [40]. To enhance the adsorption efficacy of monolayer InTe towards four SDPs (H_2_S, SO_2_, SOF_2_, and SO_2_F_2_), this study functionalizes InTe with highly reactive noble metals, Ir and Pt, by considering three distinct doping sites (Intop, Hollow, and Tetop). After full relaxation, the calculated binding energies for different doping configurations (Figure 2a) reveal that both Ir- and Pt-modified InTe systems follow the order: Hollow > Intop > Tetop. This indicates a preferential occupation of Ir and Pt atoms at the Hollow site of InTe, with E_bin_ values of −4.76 eV and −4.38 eV, respectively. Such strongly negative binding energies further confirm that the Ir and Pt atoms can be stably embedded into the InTe monolayer.

**Figure 3:**
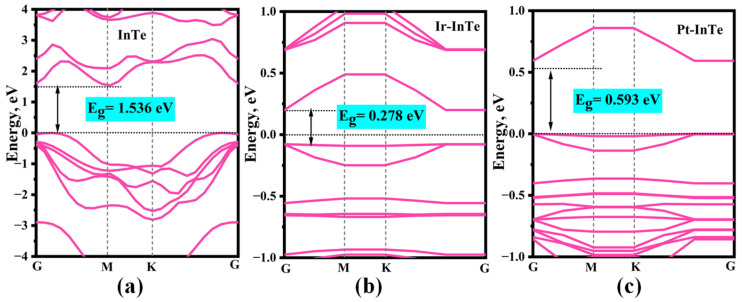
Band structures of the (**a**) pristine InTe, (**b**) Ir-InTe, and (**c**) Pt-InTe monolayers.

**Figure 4:**
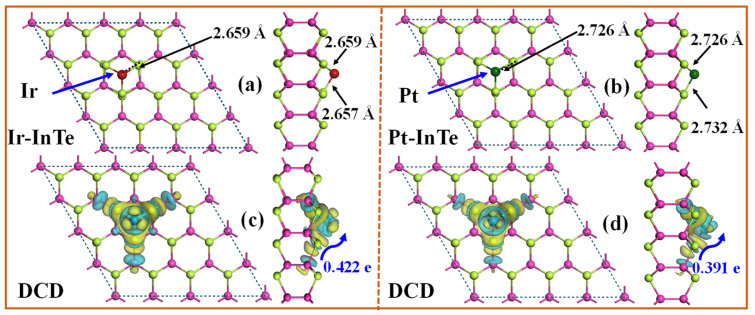
The fully relaxed structures and differential charge density (DCD) of (**a**,**c**) Ir-InTe and (**b**,**d**) Pt-InTe monolayers; the green and yellow areas denote the electrons’ gain and loss, respectively.

The band structures of the pristine InTe, Ir-InTe, and Pt-InTe monolayers are illustrated in Figure 3. Conductivity is greatly increased by this doping, which lowers the bandgap of the InTe monolayers from 1.536 eV (pristine) to 0.278 eV (Ir-doped) and 0.593 eV (Pt-doped). Figure 4 presents the lowest-energy configurations and differential charge density (DCD) of the Ir-InTe and Pt-InTe monolayers. As shown in Figure 4a,b, the bond distances between Ir/Pt atoms and their three adjacent Te atoms measure approximately 2.659 Å and 2.726 Å, respectively, which are significantly shorter than the sum of atomic radii for Ir/Pt and Te. This strongly supports the formation of Ir-Te and Pt-Te chemical bonds. From Figure 4c,d, Ir and Pt atoms gather a large number of electron-rich regions, while the InTe layer is dominated by electron-deficient regions. Moreover, Ir and Pt atoms acquire approximately 0.422 e and 0.391 e from the InTe monolayer, respectively. This substantial charge transfer illustrates the strong interaction between the dopant atoms and InTe monolayer.

To further unveil the bonding characteristics of Ir-InTe and Pt-InTe systems, the projected density of states (PDOS) was analyzed, as shown in Figure 2b,c. For the Ir-InTe system, strong orbital overlap between the Ir-d and Te-p orbitals is observed in the energy range of −5.00 eV to 2.00 eV, with pronounced resonance peaks appearing at about −3.60 eV, −2.30 eV, and −0.55 eV. This indicates the formation of strong covalent bonds between Ir and Te. Similarly, for the Pt-InTe system, a more localized orbital hybridization occurs between Pt-d and Te-p orbitals in the range of −3.75 eV to 0.50 eV, leading to the formation of Pt–Te chemical bonds. In summary, these findings demonstrate that the Ir-InTe and Pt-InTe monolayers exhibit remarkable structural stability, providing a robust foundation for their application in gas sensing systems.

### 3.2. Adsorption Characteristics of the SDPs on Ir-InTe Monolayers

**Figure 5:**
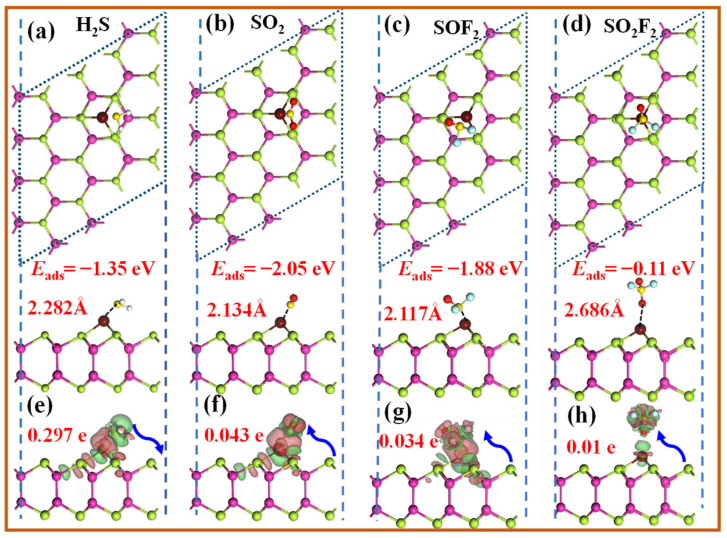
Top and side views of the optimized structures and DCD plots for the adsorbed Ir-InTe monolayers with the (**a**,**e**) H_2_S, (**b**,**f**) SO_2_, (**c**,**g**) SOF_2_, and (**d**,**h**) SO_2_F_2_. The green and yellow areas denote the electrons’ gain and loss, respectively.

This section systematically investigates the adsorption behavior of four representative SDPs on the Ir-InTe monolayer. The calculated adsorption energies, charge transfer, bandgap changes, and recovery times for Ir-InTe adsorption systems are summarized in Table 1, with their most stable adsorption configurations and corresponding DCD plots illustrated in Figure 5. For H_2_S (Figure 5a), the S atom preferentially binds to the Ir atom, yielding an *E*_ads_ of −1.35 eV and an Ir-S bond length of 2.282 Å. While SO_2_ exhibits the strongest adsorption capability on the Ir-InTe surface, with an exceptionally high *E*_ads_ of −2.05 eV and a short Ir-S bond length of 2.134 Å. Similarly, SOF_2_ shows strong chemisorption, characterized by an *E*_ads_ of −1.88 eV and an Ir-S bond length of 2.117 Å. These findings confirm that H_2_S, SO_2_, and SOF_2_ all exhibit stable chemisorption on the Ir-InTe. Conversely, the interaction between SO_2_F_2_ and Ir-InTe is obviously weak, as evidenced by its low *E*_ads_ of −0.11 eV and a longer Ir-O bond length of 2.686 Å, indicating that SO_2_F_2_ primarily undergoes weak physisorption. As shown in the DCD plots (Figure 5e–g), significant electron accumulation occurs between H_2_S, SO_2_, SOF_2_, and the Ir-InTe monolayer, highlighting their strong interactions. Specifically, H_2_S donates approximately 0.279 e to the Ir-InTe monolayer, confirming its role as an electron donor. In contrast, SO_2_ and SOF_2_ gain approximately 0.043 e and 0.034 e, respectively, from the Ir-InTe monolayer, despite their remarkably high adsorption energies. Nevertheless, SO_2_F_2_ exhibits minimal charge transfer (0.01 e), further corroborating its weak adsorption characteristics.

**Figure 6:**
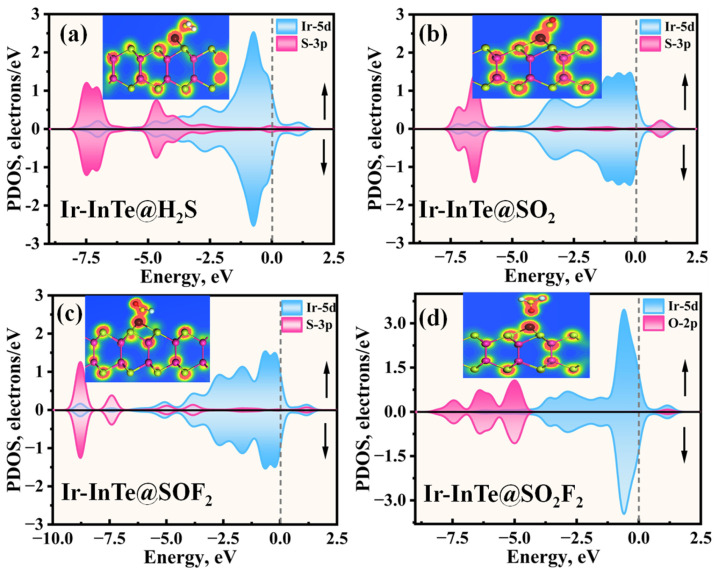
The projected density of states (PDOSs) of different adsorption systems: (**a**) Ir-InTe@H_2_S, (**b**) Ir-InTe@SO_2_, (**c**) Ir-InTe@SOF_2_, and (**d**) Ir-InTe@SO_2_F_2_, where the insets represent the TED of each system.

Figure 6 presents the PDOS for H_2_S, SO_2_, SOF_2_, and SO_2_F_2_ adsorbed on an Ir-InTe sheet. As shown in Figure 6a, significant orbital hybridization occurs between the Ir-5d and S-3p within the energy range of −8.10 eV to 1.60 eV. Furthermore, prominent overlap peaks are evident at −7.00 eV, −4.70 eV, and −2.73 eV, indicating the formation of an Ir-S covalent bond. For the Ir-InTe@SO_2_ system (Figure 6b), strong hybridization between the Ir-5d and S-3p orbitals is observed in two distinct regions: −7.50 eV to −5.50 eV and 0.00 eV to 1.30 eV, indicating a high affinity of Ir-InTe for the SO_2_. In the case of SOF_2_ adsorption (Figure 6c), the hybridization between the Ir-5d orbital and the S-3p orbital of SOF_2_ appears more delocalized, with prominent hybridization peaks at −8.80 eV, −7.40 eV, −5.00 eV, −3.80 eV, and 1.80 eV, indicating a strong interaction between SOF_2_ and Ir-InTe. Conversely, the Ir-InTe@ SO_2_F_2_ system exhibits markedly weaker hybridization, with only a minor peak near −1.25 eV, further suggesting weak physisorption.

**Figure 7:**
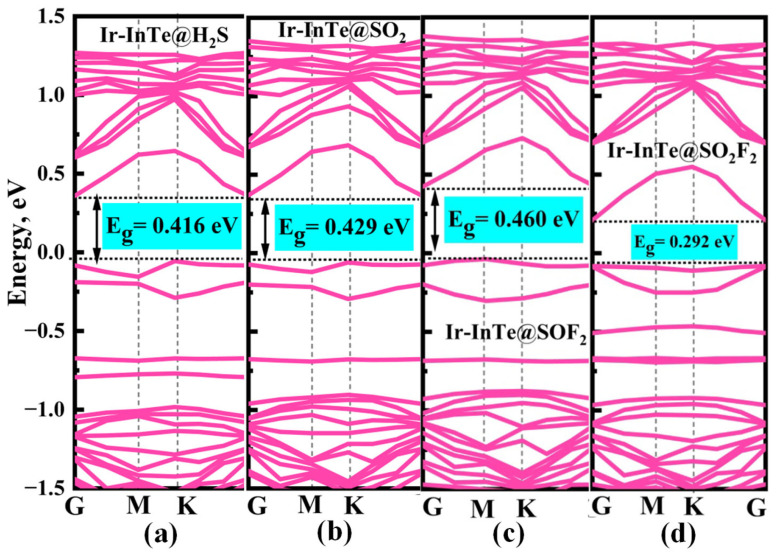
Band structures of different adsorption systems: (**a**) Ir-InTe@H_2_S, (**b**) Ir-InTe@SO_2_, (**c**) Ir-InTe@SOF_2_, and (**d**) Ir-InTe@SO_2_F_2_.

Additionally, significant charge transfer is observed between the Ir-InTe and H_2_S, SO_2_, and SOF_2_ molecules, as shown in the insets of Figure 6a–c. This observation confirms the formation of Ir–S chemical bonds in these systems. In contrast, the absence of notable charge transfer between Ir-InTe and SO_2_F_2_, as evidenced by the largely undisturbed charge density regions, indicates the lack of chemical bonding. Consequently, the adsorption strength of H_2_S, SO_2_, and SOF_2_ is substantially greater than that of SO_2_F_2_. Furthermore, the band structures of the Ir-InTe adsorption systems are presented in Figure 7. The adsorption of H_2_S, SO_2_, and SOF_2_ results in a significant increase in the bandgap of Ir-InTe, from 0.278 eV to 0.416 eV, 0.429 eV, and 0.460 eV, respectively. This pronounced bandgap modulation enhances the suitability of Ir-InTe for detecting these three gases. In contrast, the adsorption of SO_2_F_2_ has minimal effect on the bandgap due to weak physisorption.

### 3.3. Adsorption Properties of the SDPs on Pt-InTe Monolayers

**Figure 8:**
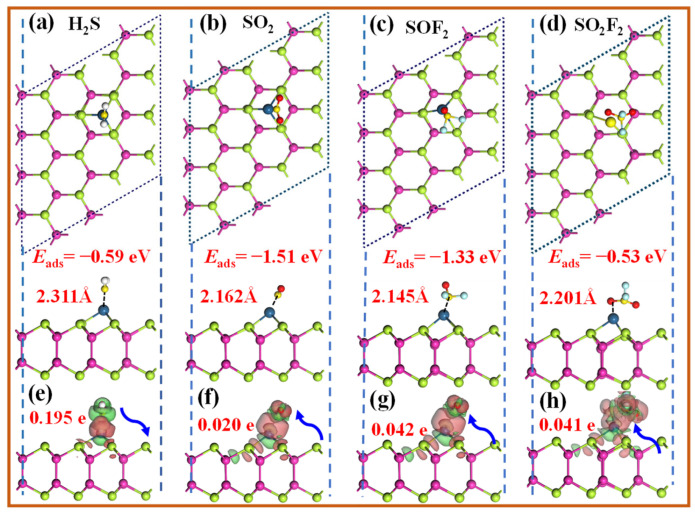
Top and side views of the optimized structures and DCD plots for the adsorbed Pt-InTe monolayers with the (**a**,**e**) H_2_S, (**b**,**f**) SO_2_, (**c**,**g**) SOF_2_, and (**d**,**h**) SO_2_F_2_. The green and yellow areas denote the electrons’ gain and loss, respectively.

The most stable adsorption configurations of the SDPs on the Pt-InTe monolayer and their corresponding DCD are illustrated in Figure 8, with the corresponding adsorption parameters listed in Table 1. As shown in Figure 8a–c, the H_2_S, SO_2_, and SOF_2_ molecules preferentially interact with the Pt atom of Pt-InTe via their S atoms. The calculated adsorption energies for H_2_S, SO_2_, and SOF_2_ are −0.59 eV, −1.51 eV, and −1.33 eV, respectively, with adsorption distances ranging from 2.162 Å to 2.311 Å. Notably, these distances are notably shorter than the sum of the covalent radii of Pt and S atoms (2.65 Å), providing strong evidence for chemisorption of these three gases. In Figure 8d, SO_2_F_2_ adsorbs above the Pt atom through its O atom, with an adsorption energy of −0.53 eV and an adsorption distance of 2.201 Å. Significantly, this adsorption distance is comparable to the sum of the atomic radii of Pt and O, indicating that SO_2_F_2_ adsorption belongs to weak chemisorption. As shown in Figure 8e, the DCD analysis reveals an electron depletion region around H_2_S and significant electron accumulation near the Pt atom on the Pt-InTe surface. This implies that H_2_S acts as an electron donor, transferring approximately 0.195 e to the monolayer. Conversely, SO_2_, SOF_2_, and SO_2_F_2_ function as electron acceptors, gaining about 0.020 e, 0.042 e, and 0.041 e from the Pt-InTe monolayer, respectively.

**Figure 9:**
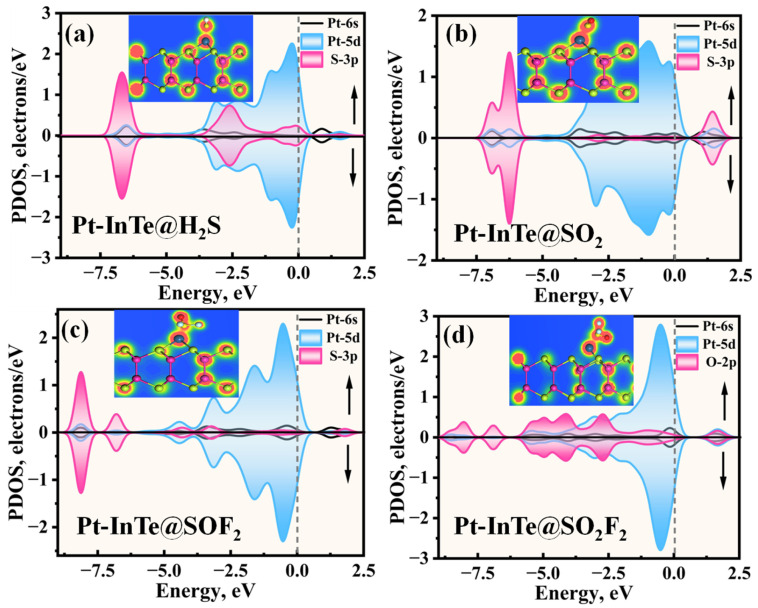
The projected density of states (PDOSs) of different adsorption systems: (**a**) Pt-InTe@H_2_S, (**b**) Pt-InTe@SO_2_, (**c**) Pt-InTe@SOF_2_, and (**d**) Pt-InTe@SO_2_F_2_, where the insets represent the TED of each system.

**Figure 10:**
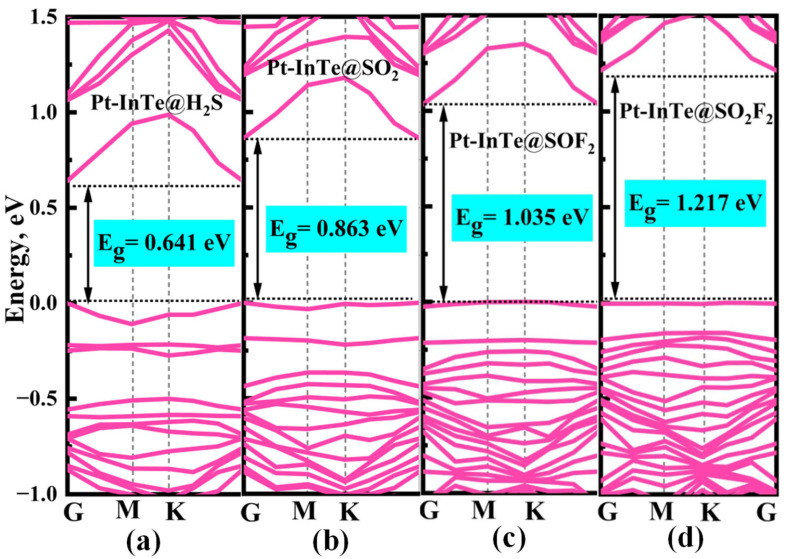
Band structures of different adsorption systems: (**a**) Pt-InTe@H_2_S, (**b**) Pt-InTe@SO_2_, (**c**) Pt-InTe@SOF_2_, and (**d**) Pt-InTe@SO_2_F_2_.

To elucidate the microscopic interaction mechanisms during adsorption, the PDOS of the Pt-InTe adsorption systems was analyzed, with results presented in Figure 9. For the H_2_S adsorption system (Figure 9a), the stable chemisorption is primarily attributed to the significant hybridization between Pt-5d and S-3p orbitals within the energy range of −7.43 eV to 2.00 eV, with three distinct hybridization peaks observed at approximately −6.50 eV, −2.60 eV, and −0.10 eV. In the SO_2_ and SOF_2_ adsorption systems, as shown in Figure 9b,c, the Pt-5d orbitals exhibit strong hybridization with the S-3p orbitals of the two gases across broader energy ranges. This robust hybridization underscores that the strong electronic interaction between SO_2_, SOF_2_, and the Pt-InTe monolayer is the primary reason for their high adsorption energies. For the Pt-InTe@SO_2_F_2_ system (Figure 9d), significant hybridization between the Pt-5d orbital and O-2p orbital of SO_2_F_2_ is evident, accompanied by distinct overlapping peaks near −6.5 eV, −4.5 eV, and −2.5 eV. Additionally, the band structures of Pt-InTe adsorption systems are depicted in Figure 10. The adsorption of H_2_S, SO_2_, SOF_2_, and SO_2_F_2_ increases the bandgaps of Pt-InTe from 0.593 eV to 0.641 eV, 0.863 eV, 1.035 eV, and 1.217 eV, respectively. This prominent bandgap modulation effect indicates a high sensitivity of Pt-InTe towards all four SDPs.

### 3.4. Gas Sensor Explorations

Sensitivity is paramount for gas sensing materials as it directly determines their ability to detect trace amounts of target gases with high precision and reliability, which is critical for applications in the detection of SDPs. The conductivity of these materials is intrinsically linked to their bandgap, and the change in conductivity can serve as the primary detection signal. Thus, the sensitivity can be quantitatively evaluated through the percentage change in bandgap (Δ*E_g_*) or work function changes (Δ*Φ*), which are defined as [36]:

(3) $$∆E_{g}={(E}_{g}^{\prime }-E_{g}^{0})/E_{g}^{0}\;\times \;100\%$$

(4) $$∆\Phi ={(\Phi }^{\prime }-\Phi^{0})/\Phi^{0}\;\times \;100\%$$

Here, $E_{g}^{0}$ and $E_{g}^{\prime }$ represent the bandgaps of sensing materials before and after gas adsorption, while $\Phi^{\prime }$ and $\Phi^{0}$ denote the work functions of sensing materials with and without gas adsorption.

**Figure 11:**
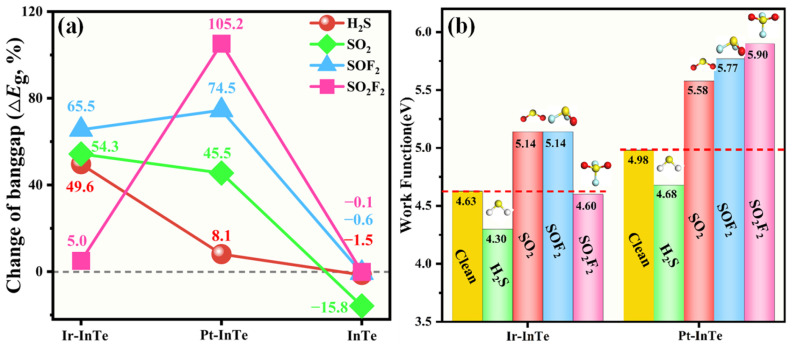
(**a**) Change in bandgap (Δ*E_g_*) and (**b**) work function values (Φ) of the pristine InTe, Ir-InTe, and Pt-InTe monolayers before and after the adsorption of SDPs.

Figure 11 illustrates bandgap and work function variations in Ir-InTe and Pt-InTe monolayers induced by the SDP adsorption. As depicted in Figure 11a, the pristine InTe monolayer exhibits poor sensitivity towards H_2_S, SOF_2_, and SO_2_F_2_ due to minimal Δ*E_g_* values, while demonstrating moderate sensitivity to the SO_2_. However, its weak SO_2_ adsorption strength limits practical applicability. Conversely, H_2_S, SO_2_, and SOF_2_ adsorption induces substantial bandgap alterations in Ir-InTe, with Δ*E_g_* values of 49.6%, 54.3%, and 65.5%, respectively, indicating that the Ir-InTe is highly sensitive to these gases. Similarly, Pt-InTe displays superior sensitivity to SO_2_, SOF_2_, and SO_2_F_2_, as evidenced by Δ*E_g_* values of 45.5%, 74.5%, and 105.2%. As shown in Figure 11b, H_2_S adsorption modulates Ir-InTe’s work function from 4.63 eV to 4.30 eV, while SO_2_ and SOF_2_ adsorption elevates it to 5.14 eV, indicating the exceptional sensitivity of Ir-InTe for these gases. Additionally, all four SDPs induce substantial changes in the work function (ΔΦ) of the Pt-InTe monolayer, ranging from −6.02% to 18.47%, thereby highlighting its potential as a work function-based sensing material. Overall, both Ir-InTe and Pt-InTe monolayers exhibit high SDP sensitivity via bandgap or work function responses.

Selectivity is another critical performance metric for gas sensors, as it defines their ability to differentiate a target analyte from interfering gases. This characteristic can be quantitatively evaluated by comparing the adsorption energy differences (Δ*E*_ads_) among various molecules. For the Ir-InTe, the adsorption energy for H_2_S (−1.35 eV) is significantly weaker than that for SO_2_ (−2.05 eV) and SOF_2_ (−1.88 eV), indicating that SO_2_ and SOF_2_ can be effectively distinguished from mixtures containing H_2_S, SO_2_, and SOF_2_. Similarly, the Pt-InTe exhibits even greater selectivity, with exceptionally large Δ*E*_ads_ values of 0.92 eV between SO_2_ and H_2_S, and 0.74 eV between SOF_2_ and H_2_S. These results highlight the excellent potential of these materials for the selective detection of sulfur oxides in complex environments. Moreover, a sensor array approach can further enhance selectivity among different SDPs. For example, while SO_2_ and SOF_2_ yield similar signals on the Ir-InTe sensor, they generate highly distinct electronic signatures on the Pt-InTe material. By integrating data from both sensors, a unique response pattern for each target gas can be established, enabling robust and reliable identification.

Recovery time is a critical performance metric for a gas-sensitive material, reflecting its ability to revert to the initial state after exposure to a target gas. According to the transition state theory, the recovery time (τ) can be calculated by [41,42]:

(5) $$\tau \;={{\;\upsilon }_{0}}^{-1}\exp(-E_{a}/k_{B}T)$$

where ${\;\upsilon }_{0}$, $E_{a}$, $k_{B}$, and T represent the attempt frequency, the adsorption energy of a system, the Boltzmann constant, and the absolute temperature, respectively. For the attempt frequency (*υ*_0_), we adopt the commonly used value of 10^12^ s^−1^, which is a standard approximation for gas desorption processes in theoretical studies [43,44].

**Figure 12:**
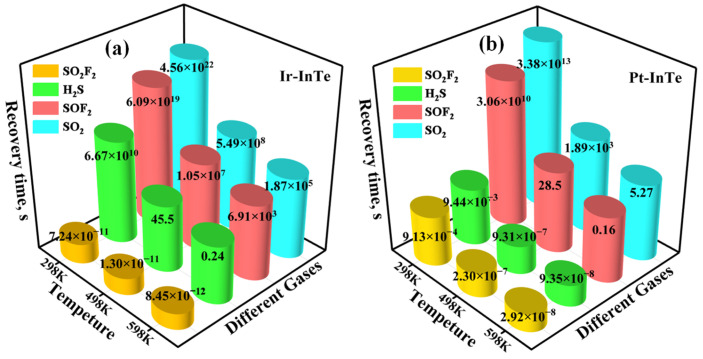
Recovery times of the H_2_S, SO_2_, SOF_2_, and SO_2_F_2_ desorbing from the (**a**) Ir-InTe and (**b**) Pt-InTe monolayers.

Figure 12 presents the recovery times of four SDPs desorbing from Ir-InTe and Pt-InTe monolayers at varying temperatures. For the Ir-InTe systems (Figure 12a), the recovery time of SO_2_F_2_ at 298 K is negligible owing to weak adsorption, which may lead to unstable electrical signals. In contrast, SO_2_ and SOF_2_ exhibit exceptionally prolonged recovery times of 4.56 × 10^22^ s and 6.09 × 10^19^ s, respectively. Even at 598 K, their recovery times remain exceptionally long, indicating significant challenges in desorbing SO_2_ and SOF_2_ from the Ir-InTe surface. Consequently, Ir-InTe holds substantial promise as a single-use gas sensing material for SO_2_ and SOF_2_ detection. Notably, Ir-InTe exhibits suitable recovery times of 45.5 s and 0.24 s for H_2_S at 498 K and 598 K, respectively, highlighting its suitability as a reusable high-temperature H_2_S sensor. In the Pt-InTe systems (Figure 12b), all four SDPs exhibit either impractically short or excessively long recovery times at 298 K, limiting room temperature reusability. However, at 598 K, Pt-InTe achieves reasonable recovery times of 5.27 s for SO_2_ and 0.16 s for SOF_2_. In summary, Ir-InTe is promising for reusable H_2_S sensing at high temperature, whereas Pt-InTe functions as a recyclable sensor for SO_2_ and SOF_2_ detection under high-temperature conditions.

## 4. Conclusions

In this work, the structural, electronic, and gas sensing properties of Ir-InTe and Pt-InTe monolayers towards four SDPs (H_2_S, SO_2_, SOF_2_, and SO_2_F_2_) were systematically explored via the use of first-principles calculations. The key findings are summarized as follows:

(1) Ir and Pt atoms stably embed into the hollow sites of the InTe monolayer, driven by strong orbital hybridization between Ir/Pt *d* and Te *p* orbitals. These modifications significantly reduce the bandgap of the InTe monolayer.
(2) The unique d-orbital configurations of Ir and Pt atoms facilitate chemisorption of SDPs. Ir-InTe exhibits strong affinity for H_2_S, SO_2_, and SOF_2_ (|*E*_ads_| ≥ 1.35 eV), while Pt-InTe effectively chemisorbs all four gases, as corroborated by the PDOS, DCD, and charge transfer analyses.
(3) Ir-InTe is highly sensitive to H_2_S, SO_2_, and SOF_2_, with bandgap change (Δ*E_g_*) values of 49.6%, 54.3%, and 65.5%, respectively, while Pt-InTe displays superior sensitivity to SO_2_, SOF_2_, and SO_2_F_2_, as evidenced by the large Δ*E_g_* values of 45.5%, 74.5%, and 105.2%. Furthermore, both monolayers exhibit notable work-function-based sensitivity to H_2_S, SO_2_, and SOF_2_.
(4) Recovery time analysis suggests that Ir-InTe is a promising reusable sensor for H_2_S, whereas Pt-InTe serves as a recyclable detector for SO_2_ and SOF_2_ under high-temperature conditions. This work provides a theoretical foundation for designing efficient InTe-based sensors for real-time SDP monitoring. To validate the predictions, future research should integrate DFT with synthesis and in situ testing.

## Figures and Tables

**Table 1:** Summary of key calculated parameters for SDPs adsorption on Ir-InTe and Pt-InTe monolayers, including adsorption energy (*E*_ads_), charge transfer (*Q*_T_), initial bandgap (*E*_g_), bandgap after adsorption (*E*′_g_), and recovery time (*τ*) at 598 K.

Materials	Gases	*E*_ads_, eV	*Q*_T_, e	*E*_g_, eV	*E′*_g_, eV	*τ*, s
Ir-InTe	H_2_S	−1.35	+0.279	0.278	0.416	0.24
	SO_2_	−2.05	−0.043	0.278	0.429	1.87 × 10^5^
	SOF_2_	−1.88	−0.034	0.278	0.460	6.91 × 10^3^
	SO_2_F_2_	−0.11	−0.010	0.278	0.292	8.45 × 10^−12^
Pt-InTe	H_2_S	−0.59	+0.195	0.593	0.641	9.35 × 10^−8^
	SO_2_	−1.51	−0.020	0.593	0.863	5.27
	SOF_2_	−1.33	−0.042	0.593	1.035	0.16
	SO_2_F_2_	−0.53	−0.041	0.593	1.217	2.92 × 10^−8^

## Data Availability

The data are not publicly available due to privacy or ethical restrictions. The data presented in this study are available on request from the corresponding author.
